# What happens to coroners’ recommendations for improving public health and safety? Organisational responses under a mandatory response regime in Victoria, Australia

**DOI:** 10.1186/1471-2458-14-732

**Published:** 2014-07-18

**Authors:** Georgina Sutherland, Celia Kemp, Lyndal Bugeja, Graham Sewell, Jane Pirkis, David M Studdert

**Affiliations:** 1 Melbourne School of Population and Global Health, University of Melbourne, Parkville, VIC, Australia; 2 Coroners Court of Victoria, 222 Exhibition St, Melbourne, VIC, Australia; 3 Department of Management and Marketing, Faculty of Business and Economics, University of Melbourne, Parkville, VIC, Australia; 4 Stanford University School of Medicine and Stanford Law School, Stanford, CA, USA

**Keywords:** Coroner, Recommendations, Public health, Organisations, Survey

## Abstract

**Background:**

Several countries of the British Commonwealth, including Australia and the United Kingdom, vest in coroners the power to issue recommendations for protecting public health and safety. Little is known about whether and how organisations that receive recommendations act on them. Concerns that recommendations are frequently ignored prompted the government of Victoria, Australia, to introduce a requirement in 2008 compelling organisations that receive recommendations to provide a written statement of action.

**Methods:**

We conducted a prospective study of organisations that received recommendations from Victorian coroners over a 33-month period. Using an online survey, we asked representatives of "recipient organisations" what action (if any) their organisations took, and what factors influenced their decision. We also probed views of the quality of the recommendations and the mandatory response regime in general. Responses were analysed at the recommendation- and recipient organisation-level by calculating counts and proportions and using chi-square analyses to test for sub-group differences.

**Results:**

Ninety of 153 recipient organisations surveyed responded (59% response rate); they received 164 recommendations (mean = 1.9; range, 1–7) from 74 cases. A total of 37% (60/164) of the recommendations were accepted and implemented, 27% (45/164) were rejected, and for 36% (59/164) the recommended action was "supplanted" (i.e., action had already been taken). In nearly half of rejected recommendations (18/45), recipient organisations indicated implementation was not logistically viable. In half of supplanted recommendations, an internal investigation had prompted the action. Three quarters (67/90) of recipient organisations believed the introduction of a mandatory response regime was a good idea, but fewer regarded the recommendations they received as appropriate (52/90) or likely to be effective in preventing death and injury (45/90).

**Conclusions:**

Only a third of coroners’ recommendations were implemented by the organisations to which they were directed. In drawing policy lessons, it is important to separate recommendations that were rejected from those in which action had already been taken. Rejected recommendations raise questions about the quality of the recommendations, the reasonableness of the organisation’s response, or both. Supplanted recommendations focus attention on the adequacy of consultation between coroners and affected organisations and the length of time it takes for recommendations to be issued.

## Background

The principal role of coroners is to investigate and determine causes of deaths that occur in sudden, unexpected or unnatural circumstances. This work advances public health indirectly, not least by enhancing the quality of vital statistics. In some countries—including Australia, New Zealand, the Republic of Ireland, the United Kingdom and most Canadian provinces—coroners are also vested with authority to play a more direct role in advancing public health: as part of their findings in particular cases, they may issue recommendations aimed at reducing risk and improving health and safety in the community [1,2].

Few studies have systematically examined coroners’ recommendations and little is known about how effectively this function is exercised. But scepticism abounds. Some commentators have questioned the appropriateness of arming coroners with recommendation powers [3-5]. Criticism has also been levelled at the practicality and evidentiary basis of some recommendations [6-9]. However, the most prevalent concern, voiced in a succession of public inquiries [10-12], is that coroners’ recommendations are ineffective because the organisations and industries to which they are directed ignore them.

The latter concern recently prompted the government of Victoria, Australia, to revamp the recommendations process as part of a wider reform of the state’s coronial system. From November 2009, an organisation that receives a recommendation from a coroner must respond in writing within three months, outlining what if any action it has taken in response [13]. There is no requirement to implement recommended action, merely to respond. However, the Coroners Court of Victoria (CCOV) must make each response publicly available via the Internet, alongside the findings and recommendations from the case. This mandatory response regime is novel. Although several other Australian jurisdictions require responses for specific types of deaths (e.g. deaths in custody), none do so across the board. To the best of our knowledge, England and Wales are the only international jurisdictions with mandatory response regimes that resemble Victoria’s, although responses in those regimes are not published [14].

Introduction of Victoria’s mandatory response regime provided an opportunity to investigate the behaviour and attitudes of organisations that respond to coroners’ recommendations. We did not rely on the organisation’s written responses, which can be unclear and non-specific. Rather, during the first several years of the new regime, we systematically surveyed organisations that received recommendations. Our main goal was to shed light on what, if any, action organisations had taken in response. A secondary goal was to probe organisations’ views about the recommendations they had received, the response process, and the mandatory response regime more generally.

## Methods

### Setting

Coroners in Victoria may issue recommendations as part of their written findings from a death investigation. The investigation may be desk-based, or involve an inquest, however most recommendations come from investigations that have gone to inquest [15]. Recommendations may be directed at any entity [16]. For simplicity, we refer to each instance of the death investigation-finding-recommendation continuum as "cases"; we refer to entities that are required to respond as "recipient organisations".

In any given case, a coroner may direct one or more recommendations to one or more recipient organisations. All combinations are possible: a recipient organisation may receive one or more recommendations, and recommendations may be directed at a single recipient organisation, or distributed across several. This heterogeneity necessitated the development of counting conventions and careful consideration of the appropriate level at which to collect and analyse data. A detailed description of our case ascertainment procedures and steps for determining the units of analysis are presented in Additional file 1.

### Study sample

We invited all organisations that received recommendations during the first 33 months (1 November 2009 to 31 July 2012) of Victoria’s new mandatory response regime to complete an online survey. Recipient organisations were eligible for inclusion in the study if they: a) had one or more recommendations directed to them; b) were mandated to respond (all "public statutory authorities and entities" are mandated, but Ministers are exempt); [13] and c) had provided a written response to the coroner. Some recipient organisations received recommendations arising from multiple cases during the study period. These "repeat players" were surveyed in relation to each case. To minimise respondent burden, however, if the most appropriate person within a recipient organisation had already been surveyed twice, they were not surveyed again.

Over the study period coroners issued recommendations in 146 cases to 228 recipient organisations. Several exclusions from this sample frame were necessary: 22 recipient organisations were not surveyed because the relevant person or unit had already completed two surveys; 13 recipient organisations had received recommendations by mistake; one case produced more than 50 recommendations directed at three recipient organisations, which were not surveyed because the recommendation-level questions in the survey would have been too onerous; two recipient organisations were not surveyed because initial contact revealed that another recipient organisation in the case had taken over responsibility for their response; and one recipient organisation from the private sector had gone into administration at the time of the survey. In addition, three organisations pre-emptively opted out of participating in the study; collectively, they received recommendations in 34 cases. These exclusions left a total of 153 recipient organisations (96 of which were unique), all of which were invited to participate in the study.

### Recruitment

We invited recipient organisations to participate in the study a minimum of six months after the coroner had handed down findings and recommendations in the case. The average time from receipt of recommendations to survey date was 13 months (SD = 6.6). Initial contact was with either the signatory to the formal response letter or a contact person named in the letter. The goal of this initial approach was to identify the most senior person in the organisation who had direct knowledge of how the organisation had responded to the coroner’s recommendation(s). Once the target respondent was identified, we forwarded a study information sheet and a hyperlink to the online survey instrument. Non-respondents were re-contacted with three reminders spaced at two-week intervals.

### Measures

We developed the survey instrument based on a review of relevant literature and input from a project Advisory Committee that included the chief coroners of Australia’s three most populous states (New South Wales, Queensland, Victoria). In addition, a draft instrument was pre-tested for content validity and comprehension with officials from two organisations that regularly receive coroners’ recommendations.

The survey asked participants, in relation to each recommendation received, to indicate if their organisation had: (i) taken action along the lines of what was recommended, (ii) taken the recommended action, but not in response to coroner’s recommendation; or (iii) not taken the recommended action. Participants then rated the importance of a range of factors in determining the nature of their response (on 5-point Likert scales ranging from "very important" to "not important"). Participants also rated their level of agreement with several statements about the relevance, appropriateness and effectiveness of both the coroners’ recommendations they received and the mandatory response regime in general (5-point Likert scale from "strongly agree" to "strongly disagree"). In addition, we queried participants about the costs of preparing their response and taking action (for those that did). Finally, for purposes of describing the sample, we extracted several variables (e.g. case dates, presiding coroner, type of death) from the written case findings.

Survey items measuring recipient organisations’ views of the recommendations and the mandatory response regime exhibited strong internal consistency (Cronbach’s alpha = 0.76 and 0.81, respectively), as did recommendation-level survey items measuring important factors in deciding whether to accept or reject recommendations (Cronbach’s alpha = 0.90).

### Analysis

Responses to questions about views of coroners’ recommendations and the mandatory response regime were analysed at the recipient organisation level, as were responses to questions about the costs of responding and taking action. All other analyses were conducted at the recommendation level.

For each recommendation, we calculated counts and proportions to describe recommendation uptake. Likert-scale responses rating importance were dichotomized (very important/important vs moderately important/of little importance/not important), as were Likert-scale responses rating level of agreement with specific aspects of the recommendation and response process (strongly agree/agree vs neither agree nor disagree/disagree/strongly disagree). "Don’t know" responses were excluded from all counts. Finally, we used chi-square analyses to test for relationships between recipient organisation’s uptake and views of recommendations. All analyses were conducted using Stata version 12 (Cary, North Carolina).

### Ethics approval

The study was approved by the human research ethics committees at the University of Melbourne and the Victorian Department of Justice.

## Results

### Sample characteristics

Participants from 90 of the 153 recipient organisations surveyed completed the online survey, a 59% response rate. Forty-three of those 90 recipient organisations responded in relation to recommendations issued in one case only; the other 47 received and responded to recommendations in multiple cases.

The 90 recipient organisations received a total of 164 recommendations (mean = 1.9; range, 1–7). These recommendations were issued in 74 coronial cases relating to 86 deaths. The recommendations varied widely in form, content and specificity. Recurrent themes included requests for changes relating to education programs, information handling practices, organisational policies and procedures, documentation, governance, public awareness activities, regulations, the built environment, and the use of preventive devices.

Characteristics of the recipient organisations and deaths are detailed in Table 1. Statutory authorities or regulators (28%), government departments (24%), and health care institutions (16%) were the most common types of recipient organisations; together they accounted for 68% of all recipient organisations. Transport (24%), complications of health care (17%), and drowning (10%) were the leading causes of death. There were no significant differences between participants and non-participants across any of the characteristics reported in Table 1.

**Table 1:** Characteristics of recipient organisations and deaths investigated

**Recipient organisation (n = 90)**	**n**	**%**
**Type**		
Statutory authority and/or regulatory agency	25	28%
Government department	22	24%
Health care institution	14	16%
Peak body/professional association	7	8%
Private company	5	6%
Local council	5	6%
Emergency service	4	4%
Custodial and justice health service	4	4%
Other	4	4%
**Size (full time equivalent employees)**		
Large (>200)	64	71%
Medium (20–200)	21	23%
Small (<20)	5	6%
**Location**		
Victoria	77	86%
Another State or Territory	1	1%
National	12	13%
**Deaths (n = 86)**	**n**	**%**
**Cause**		
Transport	21	24%
Complications of health care	15	17%
Drowning	9	10%
Natural cause	8	9%
Intentional self-harm	7	8%
Poisoning	6	7%
Falls	3	3%
Assault	3	3%
Other	14	16%

Cases were presided over by 19 different coroners working in metropolitan (n = 12) and regional (n = 7) areas of Victoria. An inquest was held in 69% (51/74) of cases and three-quarters (123/164) of all recommendations arose from cases that went to inquest. The average time elapsed between the death date and the date the recommendations were handed down was 34.8 months (SD = 17.7).

### Before the response

Fifty-nine percent (53/90) of recipient organisations reported having had an opportunity to provide information to the coroner during the investigation. A majority of this group (40/53) felt their views had been taken into account in formulation of the recommendations. For 93% (153/164) of recommendations, recipient organisations indicated that they were clear about what they were being asked to do.

### Response to recommendations

In response to 37% (60/164) of recommendations, recipient organisations implemented the recommended action (referred to hereafter as "accepted"). In 36% (59/164) of recommendations, the recommended action was taken, but not in response to the coroner’s recommendation ("supplanted"). Recipient organisations did not take the recommended action in the remaining 27% (45/164) of recommendations ("rejected").

### Reasons for accepting or rejecting recommendations

Prevention of future injury or death was cited as an important reason for taking action in relation to nearly all (56/60) of the recommendations that were accepted (Table 2, upper half). Other reasons cited as important in accepting the recommendations were the reputational implications of not acting (37/60) and awareness that a written response was mandatory and would be posted online (31/60).

**Table 2:** Reasons for accepting or rejecting coroners’ recommendations

	**Rated as important or very important**	
**Reasons for accepting recommendations (n = 60)**	**n**	**%**
Prevention of future injury or death	56	93%
Reputational implications of not acting on recommendations	37	62%
Knowing the response was mandatory and posted online	31	52%
Legal implications of not acting on coroners’ recommendations	25	42%
Knowing the recommendation was posted online	22	37%
**Reasons for rejecting recommendations (n = 45)**		
Recommendation not logistically viable	18	40%
Recommendation not relevant to business/operations	16	36%
Recommendation not economically viable	11	24%
Recommendation not clear	3	6%

Nearly half (18/45) of the recipient organisations that rejected recommendations indicated that an important reason for doing so was that the recommended action was not logistically viable (Table 2, lower half). Other reasons cited as important in the decision to reject were that the recommendation was not relevant to the organisation’s business or its operations (16/45) and the recommended action was not economically viable (11/45).

### Action taken in accepted recommendations

Among accepted recommendations, the most common types of action taken were implementation of education programs or training (36/60) and adoption or modification of policies, procedures or standards (28/60) (Table 3). For nine accepted recommendations, the recipient organisation had resolved to implement the coroner’s recommendation, but the precise form of action was still under consideration.

**Table 3:** Type of action taken in accepted recommendations* (n = 60)

	**n**	**%**
Education/training programs	36	60%
Policy/procedures/standards	28	47%
Still actively considering	9	15%
Equipment/product modification	5	8%
Behaviour modification	5	8%
Legislative change	2	3%
Other	12	20%

*Respondents could select multiple response options.

### Prompts to take action in supplanted recommendations

In nearly half (28/59) of supplanted recommendations, an investigation into the death by the recipient organisation itself was cited as the reason for having taken the action prior to the coroner’s recommendation (Table 4). The next most commonly-cited prompts were the death itself (24/59) and the coroner’s investigation (12/59).

**Table 4:** Prompts for taking recommended action, but not in response to coroners’ recommendations* (n = 59)

	**n**	**%**
Internal investigation	28	47%
The death	24	41%
Coroner’s investigation	12	20%
Anticipation of recommendations	6	10%
External investigation	4	7%
Other	19	32%

*Respondents could select multiple response options.

### Costs of response process and action taken

The estimated financial costs to recipient organisations of preparing their response ranged from zero (20/90) to over $100,000 (1/90), with 79% of recipient organisations (71/90) estimating the costs at less than $20,000. Among the recipient organisations that implemented all or some of the recommendations directed to them, 57% (42/74) estimated the financial cost of implementing the recommendation at less than $50,000 and 15% (11/74) estimated costs of between $50,000 and $500,000. One organisation estimated the cost of implementing the coroner’s recommendation at more than $1,000,000.

### Views of recommendations and the mandatory response regime

Approximately 60% of recipient organisations agreed that the recommendations they had received were useful to their organisation (54/90) or to their industry as a whole (56/90; Table 5). However, only half (45/90) believed the recommendations would be effective in preventing death and injury in the future. There was substantial support for making findings, recommendations and responses available to the public, with the vast majority of recipient organisations agreeing with the statement that it is a good idea to publish coroners’ findings and recommendations (83/90) and organisations’ responses (78/90) on the Internet. In line with their experience with recommendations, however, only about half the recipient organisations (43/90) thought the new regime would result in long-term reductions in preventable death and injury in the community.

**Table 5:** Organisations’ views of recommendations and the mandatory response regime (n = 90)

	**Agree or strongly agree**	
	**n**	**%**
**Recommendations received**		
Recommendation was useful to my industry/sector	56	62%
Recommendation was useful to my organisation	54	60%
Recommendation made to my organisation was appropriate	52	58%
Recommendation will be effective in preventing death and injury	45	50%
**Mandatory response regime**		
Publication of coroners’ findings/recommendations is a good idea	83	94%
Publication of organisations’ responses is a good idea	78	87%
The introduction of a mandatory response regime was a good idea	67	74%
The new regime will help to decrease death and injury	43	48%

Views varied systematically according to how the recipient organisation had responded to the recommendations directed to them. For example, compared to all other recipient organisations, organisations that implemented the recommendations they received were more likely to perceive them as useful (43/57 vs 11/33, p < 0.001), regard the mandatory response regime as a good idea (47/57 vs 20/33, p = 0.02), and agree that it would help to decrease death and injury (32/57 vs 11/33, p = 0.04).

## Discussion

This study found one-third of coroners’ recommendations for improving health and safety were accepted and implemented by the organisations to which they were directed. The other two-thirds were either rejected or the recommended action had already been taken. Leading reasons for rejection were that the recommendation was not logistically viable or relevant to the recipient organisation’s business; the leading prompt for taking action that pre-empted the recommendation was an internal investigation conducted by the organisation itself. Although there was broad support for the mandatory response regime as a whole, recipient organisations had mixed views about the quality and effectiveness of the specific recommendations they had received.

Despite substantial commentary on coroners’ recommendation powers and organisational responses to coroners, there is a paucity of empirical research on the topic. In a 2008 study, Watterson et al. [17] sent letters to several hundred organisations located in every Australian jurisdiction except Queensland, following their receipt of coroners’ recommendations, to inquire how they had responded. The proportion of recommendations accepted or partially accepted by organisations in Victoria (39%) was almost identical to our finding. However, a head-to-head comparison of our study findings with Watterson et al’s is not possible because they classified a large proportion of organisational responses as unknown (34% in Victoria, 18% nationally). Moreover, key aspects of Watterson et al’s methodology were not reported, including the number and type of organisations surveyed and the way in which determinations were made about implementation of coronial recommendations.

Queensland, the only state omitted from Watterson et al’s study, undertook its own review of organisational responses in 2006, focusing on recommendations made to public sector agencies [10]. The review found a relatively high rate of implementation (68% fully or partially implemented), but there was no distinction made between action taken before or after recommendations were handed down. The review did, however, elicit reasons for rejection: it found remarkably similar rationale to those detected in our study, including concerns that recommendations were too costly, inappropriate or unrealistic, or not within the organisation’s power to implement.

In our study, almost all organisations that chose to accept and implement recommendations indicated that prevention of future harm was an important rationale for doing so. Pragmatic considerations also appeared to play a role, with nearly two thirds of organisations that accepted recommendations citing the reputational consequences of not doing so as an important motivator. Concerns about reputation may have weighed particularly heavily given the regulatory backdrop in Victoria, a mandatory response regime in which recipient organisations’ statements of action are published [18].

Our finding that just over one third of recommendations were rejected by the organisations that received them resonates with previous studies and commentary suggesting that recommendations frequently do not effect change. Why were so many recommendations rejected? Virtually every rejected recommendation was considered logistically or economically unviable, or irrelevant to the recipient organisation’s business, and organisations frequently believed that following the recommendations would not produce positive public health outcomes. These reactions raise questions about the quality of some recommendations. On the other hand, it is important to acknowledge that we elicited only one side of the story and did not verify organisations’ claims about the problems with recommendations. Some rejections may have been inappropriate organisational responses to sound recommendations.

The true picture is likely to be more complicated than ascribing "fault" to one side or the other. Discord between what coroners believe organisations should do to protect health and safety and what organisations can, or are willing to do, may be attributable to a misalignment of perspectives. Coroners generally take a societal perspective in considering the desirability of preventive action. They are not well placed, however, to weigh the practicality, feasibility and cost of the recommended action from the perspective of the organisation. Organisations, particularly non-government ones, can be expected to take a private perspective on the cost-effectiveness of prevention. Thus, what is effective, even cost-effective, from a societal perspective may not be so for the organisation that faces opportunity costs and possibly also competitive disadvantages from certain investments in safety.

Discussion of these issues must be speculative without close examination of the feasibility, effectiveness and cost of coroners’ recommendations. This is a valuable topic for future research. Identifying suitable metrics and benchmarks for this type of evaluation will be challenging. But without such information, it is not possible to delineate the extent to which the rejection of coroners’ recommendations can be explained by low-quality recommendations, as opposed poor choices and obstinacy among recipient organisations.

The one third of recommendations not acted upon because the recommended action had already been taken raises a different set of issues. Apparently, neither the quality of the coroner’s recommendation nor the responsiveness of the recipient organisation are in doubt—indeed, supplanted recommendations imply a shared view of what preventive action ought to have been taken in the aftermath of an unexpected death. The pivotal question here is why coroners issued recommendations for action that had already been taken?

There are three plausible explanations. First, coroners had inadequate information at their disposal regarding organisational activities at the time recommendations were formulated. Several of our survey findings lend weight to this concern: less than two-thirds of recipient organisations reported having had an opportunity to provide information to the coroner during the investigation, and less than half felt the recommendations took their input into account.

A second explanation for the high proportion of supplanted recommendations is that recipient organisations acted in anticipation of what was coming from the coroner. We queried recipient organisations about whether such expectations motivated their pre-emptive action. Few said it did, although respondents may have understated or under-appreciated the influence of the spectre of coronial recommendations.

A third explanation for supplanted recommendations is that the investigation and findings process took too long. On average, cases in our sample took nearly three years from occurrence of the death to the handing down of findings and recommendations. (There was no statistically significant difference (*p* = 0.91) between the time to recommendations that were accepted (35 months), rejected (35 months) or supplanted (34 months)). The potential for these delays to undermine the effectiveness of coroners’ recommendation powers is well recognised [4]. It is hoped that any vital preventive action would have been implemented sooner. In addition, as the years pass, many organisations, along with the sectors and industries to which they belong, are likely to have progressed in safety consciousness and investment. Thus, quite apart from any specific action the death itself may have prompted, these secular trends may overtake whatever novelty coroners’ recommendations may once have had.

Our study had several limitations. First, despite guarantees of confidentiality, recipient organisations may have exaggerated their degree of responsiveness to coroners’ recommendations; it is reasonable to interpret the proportions of accepted and supplanted recommendations in our results as upper bounds, and the proportion of rejected recommendations as a lower bound. Second, our findings may not be generalisable to other jurisdictions in which coroners make recommendations, both elsewhere in Australia and overseas. In particular, the background presence of Victoria’s mandatory response regime is another reason for interpreting the uptake rate of recommendations we observed as a kind of "best case" scenario.

Third, the attitudinal questions asked knowledgeable individuals to convey views held by their employer. There is artificiality to this line of questioning: organisations do not hold views, people do. While such generalising may be relatively unproblematic for small organisations, large ones typically include individuals with a range of views [19]. Fourth, acceptance levels and attitudes among recipient organisations that did not respond to our survey may have differed systematically to those of respondents. The lack of any statistically significant differences between participants and non-participants in relation to type, size and location of organisation provides some reassurance on this front, although small sample sizes mean that some of these tests may have been underpowered. Finally, a population-based survey with close-ended responses has limited scope for probing the nature of and motivations for organisational behaviour [20]; more in-depth qualitative study of action taken and views held within recipient organisations would be a valuable complement to our findings.

## Conclusions

This study was not an evaluation of Victoria’s mandatory response regime. However, one ancillary benefit of such a regime is that it provides a fertile and lively environment for investigating how coroners issue recommendations and how organisations respond to them.

One third of the coroners’ recommendations we studied prompted action in the organisations to which they were directed. It is important to separate the two thirds that did not prompt action into recommendations that were rejected and those that echoed action already taken, because these two forms of "inaction" summon different policy considerations. Rejected recommendations raise questions about the quality of the coroners’ recommendation and reasonableness of the organisation’s response. Supplanted recommendations spotlight consultation processes and the considerable length of time it often takes coroners to produce recommendations.

## Competing interests

The authors have no completing interests.

## Authors’ contributions

GS (first author): Collected and analysed data and co-wrote a first draft of the manuscript. CK: Contributed to the development of the survey instrument, data interpretation and the critical revision of the manuscript. LB: Contributed to the design of the study, participated in the collection of data and critical revision of the manuscript. JP: Contributed to the design of the study and the critical revision of the manuscript. GS: Contributed to the design of the study and the critical revision of the manuscript. DS: Conceived the study, obtained funding, participated in the analysis and interpretation of data, and co-wrote the first draft of the manuscript. All authors read and approved the final manuscript.

## Pre-publication history

The pre-publication history for this paper can be accessed here:

http://www.biomedcentral.com/1471-2458/14/732/prepub

## Supplementary Material

Additional file 1 Technical case ascertainment and approach to analysis. Click here for file
