# Supplementary material for: What happens to coroners’ recommendations for improving public health and safety? Organisational responses under a mandatory response regime in Victoria, Australia
Source: BMC Public Health. 2014 Jul 18;14:732. doi: 10.1186/1471-2458-14-732 (PMC4223645; doi:10.1186/1471-2458-14-732)
Supplement: Additional file 1 — Technical case ascertainment and approach to analysis. [file 1471-2458-14-732-S1.docx]

**Appendix A: Technical case ascertainment and approach to analysis**

The study was designed to permit analyses at two levels: the recommendation and the recipient organisation. Hence, a recipient organisation that received three recommendations in a single case was queried separately about the nature of its response to each recommendation, and the answers counted as three observations in recommendation-level analyses. By contrast, some survey questions (e.g. views of the response regime) cut across a recipient organisation’s experience regardless of the number of recommendations received, and responses were collected, analysed and reported at the recipient organisation level.

For analytical purposes, the response data provided by organisations’ surveyed more than once were treated as independent observations. The rationale for this approach with recommendation-level data was that the questions pertained to different recommendations; the rationale with organisation-level data was that the recipient organisation’s views may have changed over successive cases and interactions with the coroner’s court. One consequence of this counting convention is that organisations may be counted more than once in the total number of recipient organisations.
